# Absorption Kinetics and Subcellular Fractionation of Zinc in Winter Wheat in Response to Nitrogen Supply

**DOI:** 10.3389/fpls.2017.01435

**Published:** 2017-08-18

**Authors:** Zhaojun Nie, Peng Zhao, Jia Wang, Jinfeng Li, Hongen Liu

**Affiliations:** Department of Resources and Environment, Resources and Environment College, Henan Agricultural University Zhengzhou, China

**Keywords:** absorption kinetics, nitrogen, root morphology, subcellular fractionation, zinc

## Abstract

Nitrogen (N) is critical for zinc (Zn) absorption into plant roots; this in turn allows for Zn accumulation and biofortification of grain in winter wheat (*Triticum aestivum* L.), an important food crop. However, little is known about root morphology and subcellular Zn distribution in response to N treatment at different levels of Zn supply. In this study, two nutrient solution culture experiments were conducted to examine Zn accumulation, Zn absorption kinetics, root morphology, and Zn subcellular distribution in wheat seedlings pre-cultured with different N concentrations. The results showed positive correlations between N and Zn concentrations, and N and Zn accumulation, respectively. The findings suggested that an increase in N supply enhanced root absorption and the root-to-shoot transport of Zn. Nitrogen combined with the high Zn (Zn_10_) treatment increased the Zn concentration and consequently its accumulation in both shoots and roots. The maximum influx rate (*V*_max_), root length, surface area, and volume of 14-d-old seedlings, and root growth from 7 to 14 d in the medium N (N_7.5_) treatment were higher, but the Michaelis constant (*K*_m_) and minimum equilibrium concentrations (*C*_min_) in this treatment were lower than those in the low (N_0.05_) and high (N_15_) N treatments, when Zn was supplied at a high level (Zn_10_). Meanwhile, there were no pronounced differences in the above root traits between the N_0.05_Zn_0_ and N_7.5_Zn_10_ treatments. An increase in N supply decreased Zn in cell walls and cell organelles, while it increased Zn in the root soluble fraction. In leaves, an increase in N supply significantly decreased Zn in cell walls and the soluble fraction, while it increased Zn in cell organelles under Zn deficiency, but increased Zn distribution in the soluble fraction under medium and high Zn treatments. Therefore, a combination of medium N and high Zn treatments enhanced Zn absorption, apparently by enhancing Zn membrane transport and stimulating root development in winter wheat. An increase in N supply was beneficial in terms of achieving a balanced distribution of Zn subcellular fractions, thus enhancing Zn translocation to shoots, while maintaining normal metabolism.

## Introduction

Zinc (Zn) deficiency is a common nutritional disorder in humans, affecting billions of people worldwide, particularly those in developing countries, where diets are often based on cereal grains with low Zn concentration (Welch and Graham, 2004; Cakmak et al., 2010). In China, Zn deficiency affects ~100 million people who live in rural areas (Ma et al., 2008).

In many countries, wheat is the main dietary component and the most important source of both calories and protein (Cakmak, 2008). In China, the Northern Winter Wheat Region contributes about 70% of national wheat production (Zhuang, 2003). However, Zn deficiency is common in the calcareous soils of Northern China, and this is becoming a limitation for the improvement of Zn content in wheat grain for many Chinese provinces (Liu, 1994). Thus, attempts to increase the Zn concentration of wheat grain, when crops are grown in Zn-deficient soils, is an important area of agricultural research (Bouis, 2003; White and Broadley, 2009). The breeding of novel genotypes with high grain Zn content and applying Zn-rich fertilizers are two applicable and sustainable strategies for the long-term improvement of grain Zn content (Bouis, 2003; Pfeiffer and McClafferty, 2007; Cakmak, 2008). Furthermore, studies report that an increase in nitrogen (N) supply to plants shows potential for agronomic Zn biofortification of wheat (Cakmak et al., 2010; Xue et al., 2012). Furthermore, N combined with Zn supply is more effective in increasing grain Zn content than single N or Zn supply, especially when wheat is cultured in Zn deficient soils. Kutman et al. (2010) pointed out that both soil and foliar N application improves Zn concentration in durum wheat grain when Zn supply is adequately high. Li et al. (2015) also report that foliar Zn combined with N increased Zn concentration and bioavailability in wheat grain during a 2-year field experiment. These studies suggest that a combined N/Zn application might be a promising strategy for increasing grain Zn concentrations to address dietary Zn deficiency.

Zinc concentration in wheat grain depends on physiological processes in plants, such as root uptake, root-to-shoot transport, phloem loading, remobilization of Zn from the source tissues into developing seeds, and deposition of Zn in the seed (Kutman et al., 2010). Grain proteins are reported to be contributed for the accumulation of Zn as there are highly positive correlations between seed protein and seed Zn (Peleg et al., 2008). Increasing the levels of N nutrition has been reported to enhance root uptake, root-to-shoot translocation, and remobilization of Zn in wheat (Cakmak et al., 2010; Erenoglu et al., 2011), while also increasing protein concentration in the grain. Meanwhile, a high N supply leads to the long term uptake of Zn resulting in increased Zn accumulation in grain, due to delayed senescence and thus an extended grain-filling period (Yang and Zhang, 2006).

The distribution of Zn at subcellular levels in plant tissues has received significant attention (Rathore et al., 1972; Chardonnens et al., 1999; Li et al., 2006; Pan et al., 2016), due to the importance of the subcellular localization of Zn relative to its functional role in cellular activities (Whatley et al., 1951). However, all these studies were carried out under conditions of excessive Zn. Plants possess a range of detoxifying cellular mechanisms, such as storing Zn within cell walls or leaf vacuoles, which result intolerance to Zn stress (Li et al., 2006). Meanwhile, little information is currently available on the subcellular distribution of Zn in plants that are cultured under conditions of deficient or sufficient Zn supply, especially combined with a varied N supply.

Excessive N fertilization in intensive agricultural areas results in severe environmental problems such as eutrophication, increased greenhouse gas emissions, and soil acidification, and has recently received greater attention in China (Zheng et al., 2004; Guo et al., 2010; Le et al., 2010). Policies related to increased levels of N application have been highlighted by the Department of Agriculture of China, in relation to the key target of balancing crop production and environmental protection.

Our previous studies showed that adequate N supply increases grain yield, total Zn accumulation, and Zn concentration in the different physiological parts of winter wheat under field conditions; of particular interest is the greater accumulation of Zn in grains as compared with other parts (Zhao et al., 2013, 2016). The aim of this study was to: (i) re-examine the influence of different levels of N and Zn application on Zn accumulation; (ii) to investigate Zn absorption kinetics influenced by different levels of N supply; and (iii) to investigate root morphology and Zn subcellular distribution in response to different levels of N supply, combined with Zn supply during hydroponic trials under greenhouse conditions.

## Materials and methods

### Greenhouse conditions

All experiments were performed in a greenhouse under controlled environmental conditions, using a light/dark regime of 14/10 h, corresponding air temperatures of 22/18°C, a photon flux density of ~500 μmol m^−2^ s^−1^, and a relative humidity of ~65%.

### Solution culture

Winter wheat (*Triticum aestivum*. cv Yunong202) seeds were disinfected in a solution of 0.5% NaClO before being germinated in deionized water (resistivity >18.25 MΩ.cm at 25°C) at 25°C for 5 d. After germination, the seedlings were transferred to 4 L plastic containers of nutrient solutions consisting of 2.5 mM K_2_SO_4_, 1 mM KH_2_PO_4_, 2 mM MgSO_4_·7H_2_O, 100 μM ethylene diamine tetraacetic acid (EDTA)-Fe, 46 μM H_3_BO_3_, 9 μM MnCl_2_·4H_2_O, 0.3 μM CuSO_4_·5H_2_O, and 0.02 μM (NH_4_)_6_Mo_7_O_24_·4H_2_O. The final pH of the solution was adjusted to 6.0. Different amounts of Zn were added in the form of ZnSO_4_·7H_2_O, depending on the experimental or treatment group, and different N supplies were established through the addition of Ca(NO_3_)_2_·4H_2_O. Low N and medium N pots were supplemented with CaCl_2_·2H_2_O to ensure adequate Ca concentrations. Quarter-strength and half-strength nutrient solutions were used during the first and second weeks, respectively. Subsequently, full-strength solutions were used until all seedlings were sampled. The solutions were refreshed every 3 d. All vessels used in the experiment were dipped in 5% HCl for 1 week prior to use and were then washed with deionized water a minimum of three times. The water used for the preparation of nutrient solutions was deionized, and all chemical reagents were of analytical grade.

### Absorption kinetics and subcellular fractionation experiments

In the first experiment, 20 seedlings per pot were cultured in a nutrient solution with 0.5 mM (N_0.5_, low), 7.5 mM (N_7.5_, medium), or 15 mM (N_15_, high) N as well as 0 μM (Zn_0_, without), 1 μM (Zn_1_, medium), or 10 μM (Zn_10_, high) Zn supply. The roots of two seedlings were sampled and analyzed for morphological characteristics after cultivation for 7 and 14 d, respectively. The shoots and roots of 14 plants were separately harvested after cultivation for 21 d, oven-dried at 65°C and analyzed for dry weights and elemental concentrations. The separated parts of other wheat plants, comprising leaves, stems, and roots, were immediately frozen in liquid nitrogen and stored at -20°C for further subcellular fractionation analysis.

The second experiment was a time course depletion experiment in which seedlings were pre-cultured in a nutrient solution for 21 d without Zn but with N_7.5_. To study the effects of varied N supply on the depletion of Zn from the nutrient solution, plants were supplied with N_0.5_, N_7.5_, or N_15_ for the next 24 h. To start the depletion experiment, the roots of the seedlings were washed in a solution containing 0.5 mM CaSO_4_ and 2 mM 2- (N- morpholine) ethanesulfonic acid (MES) for 30 min and then transferred to flasks containing 300 mL of the absorption solution, which contained macronutrients, micronutrients, and 10 μM ZnSO_4_·7H_2_O. The Zn concentration in 3 mL of nutrient solution was measured at 10 different time points (0, 0.5, 1, 2, 4, 6, 8, 10, 12, and 24 h). Throughout the experiment, the volume of solution in the pots was kept constant by adding the respective Zn-free nutrient solutions.

### Analysis of root morphological characteristics

Root length, root surface area, root volume, and average root diameter were determined from root images, using the root imaging analysis software WinRHIZO Version 2009 PRO (Regent Instruments, Quebec City, Canada).

### Tissue fractionation

Frozen materials were pretreated according to the method described by Weigel and Jäger (1980) and Li et al. (2006). A 2-g portion of frozen leaf, stem, or root was placed into 50-mL polypropylene centrifuge tubes and homogenized in 20 mL extraction buffer (50 mM Tris-HCl (pH 7.5), 250 mM sucrose, and 1.0 mM dithioerythritol). The homogenate was centrifuged at 300 × g for 30 s and the residue constituted the cell wall fraction. The supernatant was then centrifuged at 10,000 × g for 30 min and the retained pellet formed the cell organelle fraction. The resultant supernatant solution (referred to as the soluble fraction, consisting mostly of vacuoles) was used in subsequent characterization studies as described. All steps were performed at 4°C.

### Mineral analysis

For the Zn analysis, dry samples were ground and digested with 5 mL of HNO_3_: HClO_4_ (4:1, v/v). The cell wall and cell organelle fractions of tissues were transferred to 100 mL Erlenmeyer conical flasks containing deionized water, evaporated to dryness, and subjected to acid digestion as described above. The digested samples were diluted to 25 mL with deionized water and concentrations of Zn were determined by flame atomic absorption spectrophotometer (ZEEnit 700, Analytik Jena AG, Germany). The soluble fraction and solutions sampled from the depletion experiment were acidified with HNO_3_, and then determined using a flame atomic absorption spectrophotometer. For N analysis, dried and ground samples were digested with H_2_SO_4_ and H_2_O_2_, and the sample solution was determined for total N concentrations according to the Kjeldahl method, using a nitrogen autoanalyzer (BRAN LUEBBE AA3 Autoanalyzer, Germany). Measurements were checked for accuracy of Zn and N determination using certified standard reference materials, purchased from the National Center of Standard Material in China.

### Calculations and statistical analyses

Zinc and N accumulation (μg plant^−1^ and mg plant^−1^, respectively) were calculated from Zn and N concentrations, respectively, multiplied by the dry weight.

Zinc absorption kinetics were estimated using a modified Michaelis–Menten model (Barber, 1979):

(1) $$I_{n}=\frac{V_{\max}(C_{s}-C_{\min})}{K+(C_{s}-C_{\min})}$$

where: *I*_n_ is the inflow rate at substrate concentration *C*_s_; *C*_s_ is the substrate concentration in the root medium; *V*_max_ is the maximum influx rate at saturating substrate concentration; *C*_min_ is the minimum equilibrium concentration, meaning the substrate concentration in solution at which there is no net inflow (*I*_*n*_ = 0); and *K*_m_ is the Michaelis constant, equaling *C*_s_-*C*_min_, where *I*_n_ is ½ of *V*_max_. The kinetic parameters of Zn including *V*_max_ (μmol g^−1^ root fresh weight [FW]h^−1^), *K*_m_ (μmol L^−1^), and *C*_min_ (μmol L^−1^) were calculated based on the Zn depletion curve according to the method used by Jiang et al. (1995). Firstly, a quadratic curve between times and Zn concentration in solution is fitted using the non-linear regression procedure of Excel 2007:

(2) $$Y=a+bX+cX^{2}$$

where: *Y* represents Zn concentrations in solution at each time; and *X* represents the depletion time. A negative derivative equation is then obtained through Equation (2):

(3) Y′=−b−2cX

When *X* = 0, *Y*′ equals *-b*, meaning the maximum change rate of the Zn concentrations in solution. Thus, *V*_max_ is obtained through calculation:

(4) $$V_{\max}=\frac{-b\times v}{m}$$

where: v is the volume of absorption solution; and m is the weight of dry root. When *Y*′ equals –*b*/2 in Equation (3), then X_1_ equals –*b*/4*c*. *K*_m_ is calculated by substituting the above X_1_ into the Equation (2). Finally, when *Y*′ equals zero in Equation (3), then X_2_ equals –*b*/2*c*. *C*_min_ is also calculated by substituting the above X_2_ into the Equation (2).

The subcellular fractions of Zn were calculated as a percentage of total Zn in all fractions.

The significance of the effects of treatment and their interaction on the reported traits was evaluated using one-way or two-way analysis of variance (ANOVA), depending on the experimental design. The data are presented as the average of three replicates. Significant differences among means were determined, using Fisher's protected least significant difference (LSD) test at a 5% level (*P* < 0.05).

## Results

### Dry weight of shoots and roots and root-to-shoot ratios

According to the results of two-way ANOVA, not only the N and Zn treatments, but also their interaction exerted significant effects (*P* < 0.05) on the dry weight of shoots and roots (Table S1). An increase in N supply significantly increased shoot and root dry weights at each Zn treatment, with the highest value in N_15_ (Table 1). However, the root-to-shoot ratio significantly decreased with increasing N supply. Increasing Zn supply significantly increased shoot dry weight in the N_7.5_ and N_15_ treatments, and root dry weight in the N_15_ treatment. When N was supplied at rates of N_0.5_ or N_7.5_, the root dry weights also increased with the Zn_1_ treatment. Root-to-shoot ratios were significantly increased by the N_0.5_Zn_1_ treatment, while the N_7.5_Zn_10_ treatment significantly decreased root-to-shoot ratios.

**Table 1:** Shoot and root dry weights and root-to-shoot ratios of winter wheat (*Triticum aestivum* cv Yunong202) seedlings, pre-cultured with 0.5, 7.5, or 15 mmol N L^−1^ in a nutrient solution with 0, 1, and 10 μmol Zn L^−1^ supply for 21 d.

**N supply**	**Shoot (g plant**^**−1**^**)**			**Root (g plant**^**−1**^**)**			**Root: shoot**		
	**Zn_0_**	**Zn_1_**	**Zn_10_**	**Zn_0_**	**Zn_1_**	**Zn_10_**	**Zn_0_**	**Zn_1_**	**Zn_10_**
N_0.5_	0.29d	0.31d	0.30d	0.16d	0.20bc	0.16d	0.56b	0.63a	0.54b
N_7.5_	0.47c	0.60b	0.65b	0.17d	0.22b	0.17cd	0.37c	0.36c	0.27d
N_15_	0.61b	0.74a	0.76a	0.20b	0.25a	0.25a	0.33cd	0.33cd	0.33cd

*Values are means of three independent replicates. For each trait, means followed by different letters are significantly different from each other according to two-way ANOVA followed by least significant difference (LSD) multiple comparison (P < 0.05)*.

### Zn and N concentrations and their accumulation

**Figure 1:**
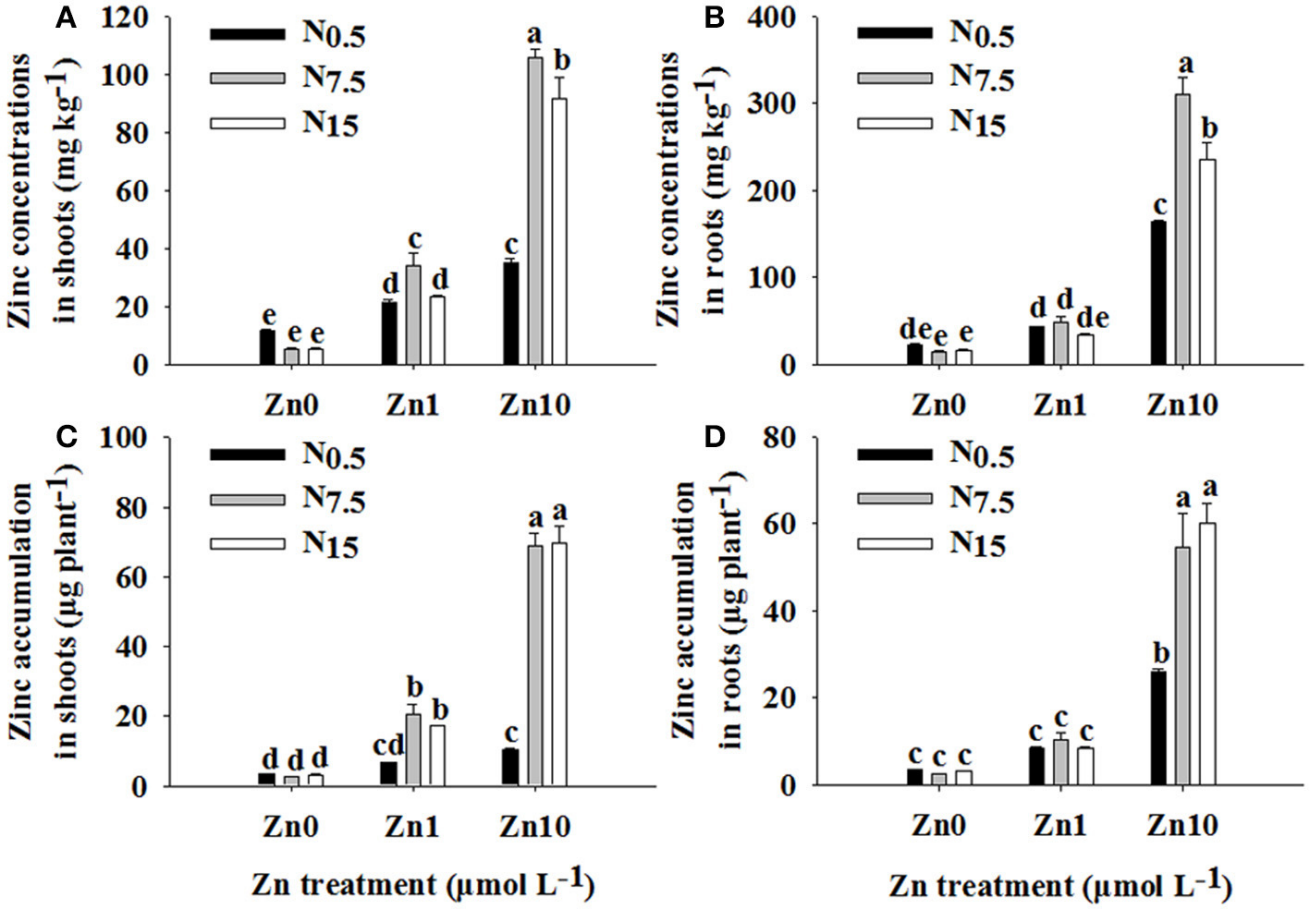
Zinc concentrations in shoots **(A)** and roots **(B)** as well as Zn accumulation in shoots **(C)** and roots **(D)** of winter wheat (*Triticum aestivum* cv Yunong202) seedlings grown at 0.5, 7.5, or 15 mmol N L^−1^ in a nutrient solution with 0, 1, and 10 μmol Zn L^−1^ supply. Values are means of three independent replicates. Error bars represent 1 SE. For each trait, means followed by different letters are significantly different from each other according to two-way ANOVA followed by least significant difference (LSD) multiple comparison (*P* < 0.05).

Two-way ANOVA revealed significant interactive effects of N and Zn treatment on Zn concentrations and its accumulation in shoots and roots (*P* < 0.01; Table S1). For each N treatment, increasing the Zn supply led to higher Zn concentrations and accumulation in shoots and roots. However, under Zn deficiency, increasing the N supply had no apparent effect on Zn concentration and accumulation in shoots and roots (Figure 1). In both Zn_1_ and Zn_10_, Zn concentration and accumulation in shoots were significantly increased by an increase in N supply (Figures 1A,C). Only in the Zn_10_ treatment did an increase in N supply significantly increase Zn concentration and accumulation in roots (Figures 1B,C).

**Figure 2:**
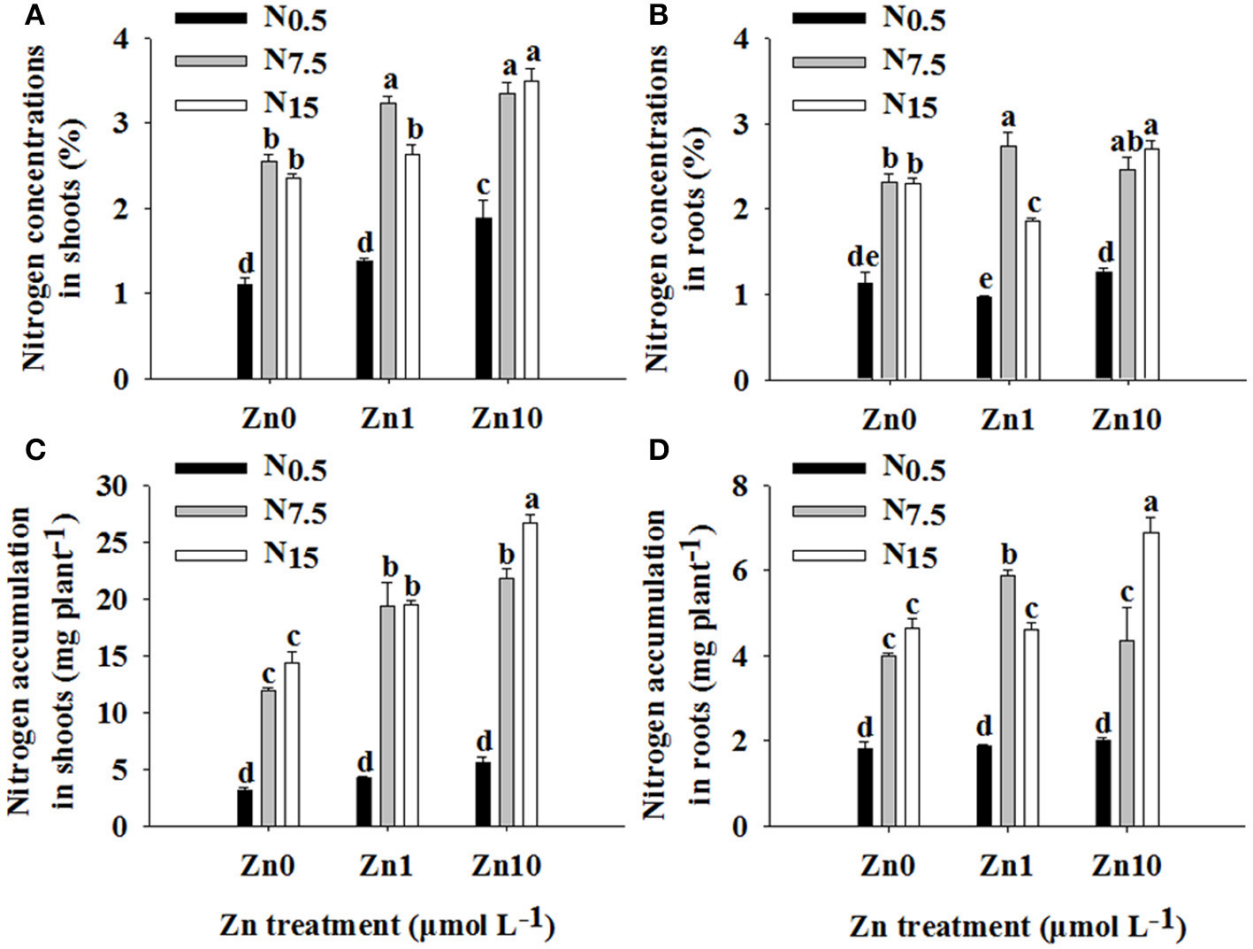
Nitrogen concentrations in shoots **(A)** and roots **(B)** and N accumulation in shoots **(C)** and roots **(D)** of winter wheat (*Triticum aestivum* cv Yunong202) seedlings grown at 0.5, 7.5, or 15 mmol N L^−1^ in a nutrient solution with 0, 1, and 10 μmol Zn L^−1^ supply. Values are means of three independent replicates. Error bars represent 1 SE. For each trait, means followed by different letters are significantly different from each other according to two-way ANOVA followed by least significant difference (LSD) multiple comparison (*P* < 0.05).

The N and Zn treatments interacted significantly in their effects on N concentration and accumulation in shoots and roots (*P* < 0.01; Table S1). An increase in N supply resulted in marked increases in N concentration and accumulation in shoots and roots (Figure 2). At N_0.5_, only the Zn_10_ treatment significantly increased shoot N concentration. At N_7.5_, an increase in Zn supply significantly increased shoot N concentration and accumulation, and only the Zn_1_ treatment significantly increased root N concentration and accumulation. In N_15_, the Zn_10_ treatment resulted in higher shoot and root N concentrations and accumulation, while the Zn_1_ treatment significantly increased shoot N accumulation but decreased root N concentration.

There were significant positive relationships between shoot N and Zn concentration (*r* = 0.647^**^), shoot N and Zn accumulation (*r* = 0.761^**^), root N and Zn accumulation (*r* = 0.445^*^), and plant N and Zn accumulation (*r* = 0.674^**^), respectively.

### Zn absorption kinetics

**Figure 3:**
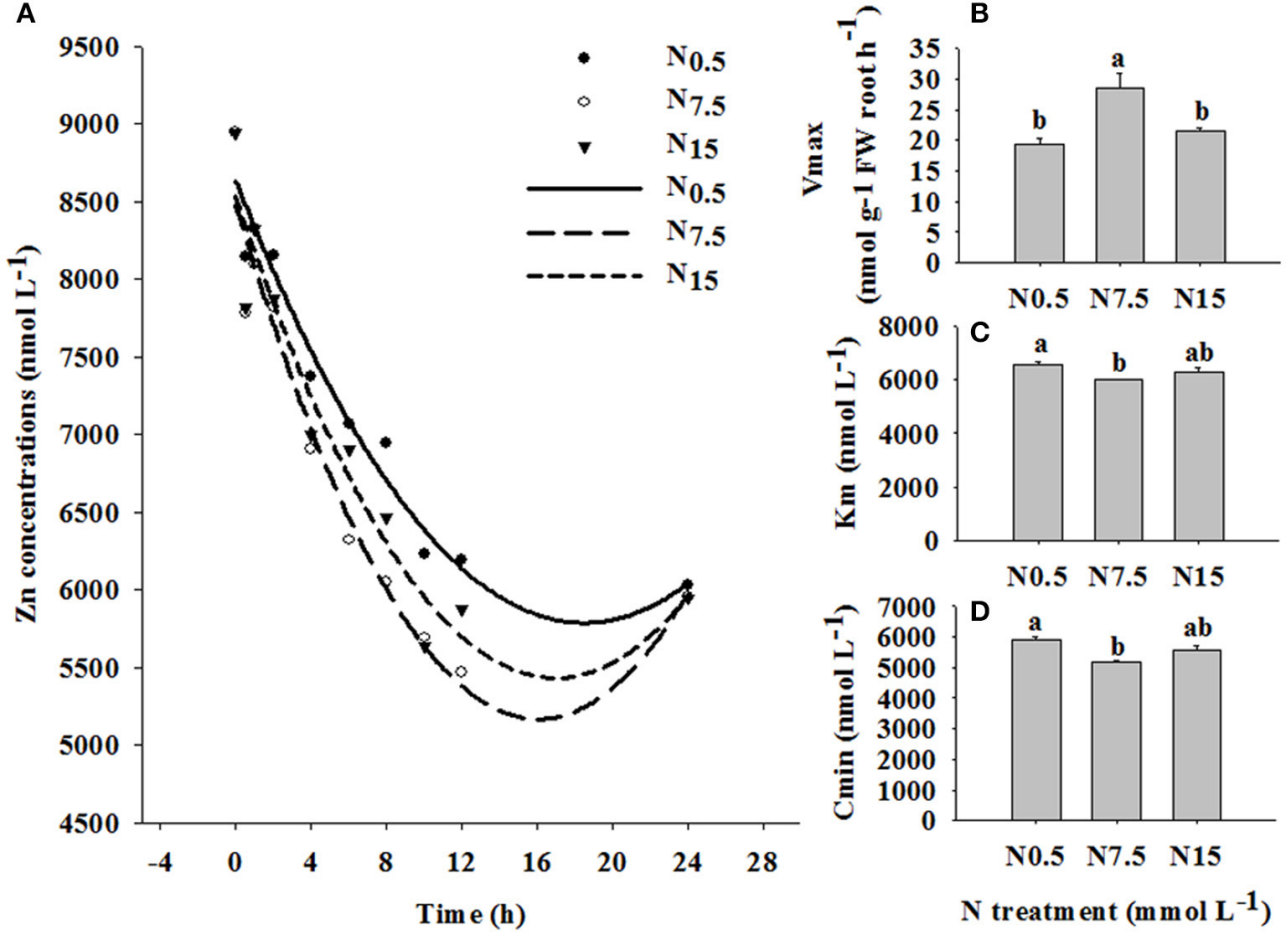
Zinc depletion in solutions containing three winter wheat (*Triticum aestivum* cv Yunong202) seedlings, grown without Zn and with medium (7.5 mmol L^−1^) N supply for 21 d and then transferred to solutions supplemented with 10 μmol Zn L^−1^ and low (0.5 mmol L^−1^; filled circles), medium (7.5 mmol L^−1^; open circles), or high (15 mmol L^−1^; triangles) N supply for varying durations (0–24 h). **(A)**: Zn depletion curve, **(B)**: maximum influx rate *V*_max_, **(C)**: Michaelis constant *K*_m_, **(D)**: minimum equilibrium concentration *C*_min_. Values are means of three independent replicates. Error bars represent 1 SE. Means followed by different letters are significantly different from each other according to one-way ANOVA followed by least significant difference (LSD) multiple comparison (*P* < 0.05).

In the time-course experiment, the depletion of Zn by plants was followed for 24 h under the three N treatments. N_7.5_ resulted in the highest level of Zn exhaustion in the solution after 1 h, followed by N_15_; N_0.5_ was the slowest to deplete Zn in the solution (Figure 3A). The values of *V*_max_, *K*_m_, and *C*_min_ were strongly dependent on the level of N supply (*P* < 0.05; Table S2). The value of *V*_max_ was higher, and the values of *K*_m_ and *C*_min_ were lower in N_7.5_, compared to N_0.5_ and N_15_ (Figures 3B,C).

### Root morphology

As revealed by two-way ANOVA, the root length, root surface area, root volume, and root average diameter of 7- and 14-d-old seedlings were significantly affected by N and Zn treatments, and N × Zn interaction (*P* < 0.05 or *P* < 0.01; Table S3). In the 7-d-old seedlings, the N_7.5_ and N_15_ treatments exhibited a significantly decreased root length for each Zn treatment (Table 2). The root surface area was also significantly decreased by N_7.5_ and N_15_ in the Zn_1_ treatment, and decreased by N_15_ in the Zn_10_ treatment. The N_15_ treatment exhibited a larger root volume under Zn deficiency, but had a smaller root volume than N_0.5_ and N_7.5_ under the Zn_1_ and Zn_10_ applications. The N_7.5_ and N_15_ treatments significantly increased root average diameter with the Zn_0_ and Zn_10_ applications, compared to the N_0.5_ treatment. The Zn_10_ treatment significantly increased root length, root surface area, and root volume in N_0.5_ and N_7.5_. The root surface area, volume, and average diameters were significantly increased by Zn_1_ with the N_0.5_ application, but decreased by Zn_1_ and Zn_10_ in the N_15_ application. In the 14-d-old seedlings, when compared to N_0.5_, the N_7.5_ treatment significantly increased length, surface area, and volume of roots at Zn_10_, while no pronounced effects appeared on the above root morphology traits in Zn_0_ and Zn_1_ (Table 2). The length, surface area, and volume of roots in N_15_ were lower than those in N_0.5_ and N_7.5_ at Zn_0_ or Zn_1_. Increasing Zn supply significantly decreased root length, surface area, and volume at N_0.5_ or N_15_. However, N or Zn treatment had no significant effect on the average root diameters of winter wheat.

**Table 2:** Root morphology parameters of winter wheat (*Triticum aestivum* cv Yunong202) seedlings after 7 and 14 d, at 0.5, 7.5, or 15 mmol N L^−1^ in a nutrient solution with 0, 1, and 10 μmol Zn L^−1^ supply.

**Growing days**	**N supply**	**Root length (cm)**			**Root surface area (cm**^**2**^**)**			**Root volume (cm**^**3**^**)**			**Average root diameter (cm)**		
		**Zn_0_**	**Zn_1_**	**Zn_10_**	**Zn_0_**	**Zn_1_**	**Zn_10_**	**Zn_0_**	**Zn_1_**	**Zn_10_**	**Zn_0_**	**Zn_1_**	**Zn_10_**
7d	N_0.5_	538bc	555b	625a	45.6cde	52.5ab	54.1a	0.31b	0.40a	0.38a	0.27c	0.30b	0.27c
	N_7.5_	465de	416e	550b	41.4de	42.9de	51.2abc	0.29b	0.41a	0.38a	0.30b	0.31ab	0.30b
	N_15_	483cd	452de	505bcd	47.4bcd	41.4de	39.9e	0.37a	0.29b	0.29b	0.32a	0.29b	0.29b
14d	N_0.5_	1329ab	1116bc	717e	122a	99.8b	70.2d	0.88a	0.79ab	0.55e	0.29bcd	0.30bcd	0.32abc
	N_7.5_	1345ab	1394a	1212abc	117a	106ab	108ab	0.80ab	0.72bc	0.77b	0.27d	0.27d	0.28cd
	N_15_	1010bc	809de	735e	93.3bc	81.3cd	74.7d	0.66cd	0.65cd	0.62de	0.32ab	0.30bcd	0.34a

*Values are means of three independent replicates. For each trait, means followed by different letters are significantly different from each other according to two-way ANOVA followed by least significant difference (LSD) multiple comparison (P < 0.05)*.

### Zn subcellular fraction and distribution

Zinc concentrations of each tissue fraction were positively affected by both the N and Zn treatments, according to the results of two-way ANOVA (*P* < 0.05 or *P* < 0.01; Table S4). Zinc concentration in each fraction of tissue showed a strong increase with increasing Zn supply, with the highest in the Zn_10_ treatment. Zinc concentration in cell walls and cell organelles of roots were significantly raised by increasing N supply at the Zn_10_ level. At Zn_0_ and Zn_1_, Zn concentrations in root cell walls and cell organelles increased when N supply increased from N_0.5_ to N_7.5_, but decreased when N supply increased from N_7.5_ to N_15_. The N_7.5_ treatment had the highest concentrations of Zn in root cell walls and cell organelles (Table 3). An increase in N supply resulted in significant increases in Zn concentration in the soluble fractions of roots for each Zn treatment. For stems of winter wheat, the Zn concentration in cell walls and cell organelles showed a strong decrease in Zn_0_ and Zn_1_ treatments with an increase in N supply. However, Zn concentration in cell walls showed a strong increase in Zn_10_ treatments with an increase in N supply; the N_7.5_ treatment exhibited higher Zn concentrations in cell organelles than the N_0.5_ and N_15_ treatments with the Zn_10_ application. An increase in N supply led to higher Zn concentrations in the soluble fraction in the Zn_1_ treatment. Zinc concentration in leaf cell walls and cell organelles were increased by an increase in N supply for each Zn treatment. An increase in N supply decreased Zn concentration in the soluble fraction with Zn_0_; however, concentration was increased in Zn_10_.

**Table 3:** Subcellular fractionation of Zn in tissues of winter wheat (*Triticum aestivum* cv Yunong202) seedlings, grown at 0.5, 7.5, or 15 mmol N L^−1^ in a nutrient solution with 0, 1, and 10 μmol Zn L^−1^ supply for 21 d.

**Tissues**	**N supply**	**Cell wall fraction (mg kg^−1^ FW)**			**Cell organelle fraction (mg kg^−1^ FW)**			**Soluble fraction (mg kg^−1^ FW)**		
		**Zn_0_**	**Zn_1_**	**Zn_10_**	**Zn_0_**	**Zn_1_**	**Zn_10_**	**Zn_0_**	**Zn_1_**	**Zn_10_**
Root	N_0.5_	6.30bc	9.42b	11.1b	1.83e	3.85c	7.15b	0.50e	0.67de	2.71c
	N_7.5_	10.6b	8.35bc	21.0a	2.54de	3.78cd	13.0a	0.94de	1.43de	11.3b
	N_15_	4.52c	7.16bc	17.8a	1.49e	1.72e	8.11b	1.55d	3.31c	13.1a
Stem	N_0.5_	10.7bc	20.9a	9.26c	4.50c	6.14b	6.38b	1.10de	1.75c	3.41a
	N_7.5_	11.4bc	11.4bc	11.9bc	2.75d	6.22b	8.45a	1.24cde	2.40b	3.92a
	N_15_	3.27d	9.11c	13.1b	2.02d	4.69c	6.87b	0.65e	1.63cd	3.94a
Leaf	N_0.5_	1.91de	2.88d	9.54ab	2.13de	1.40e	2.29de	0.57bc	0.73bc	0.80bc
	N_7.5_	1.64e	4.96c	9.08b	7.90a	2.12de	5.74bc	0.52bc	1.41bc	3.15a
	N_15_	2.63de	4.26c	10.3a	6.73ab	1.20e	4.03cd	0.25c	1.56b	2.96a

*Values are means of three independent replicates. For each trait, means followed by different letters are significantly different from each other according to two-way ANOVA followed by least significant difference (LSD) multiple comparison (P < 0.05)*.

**Figure 4:**
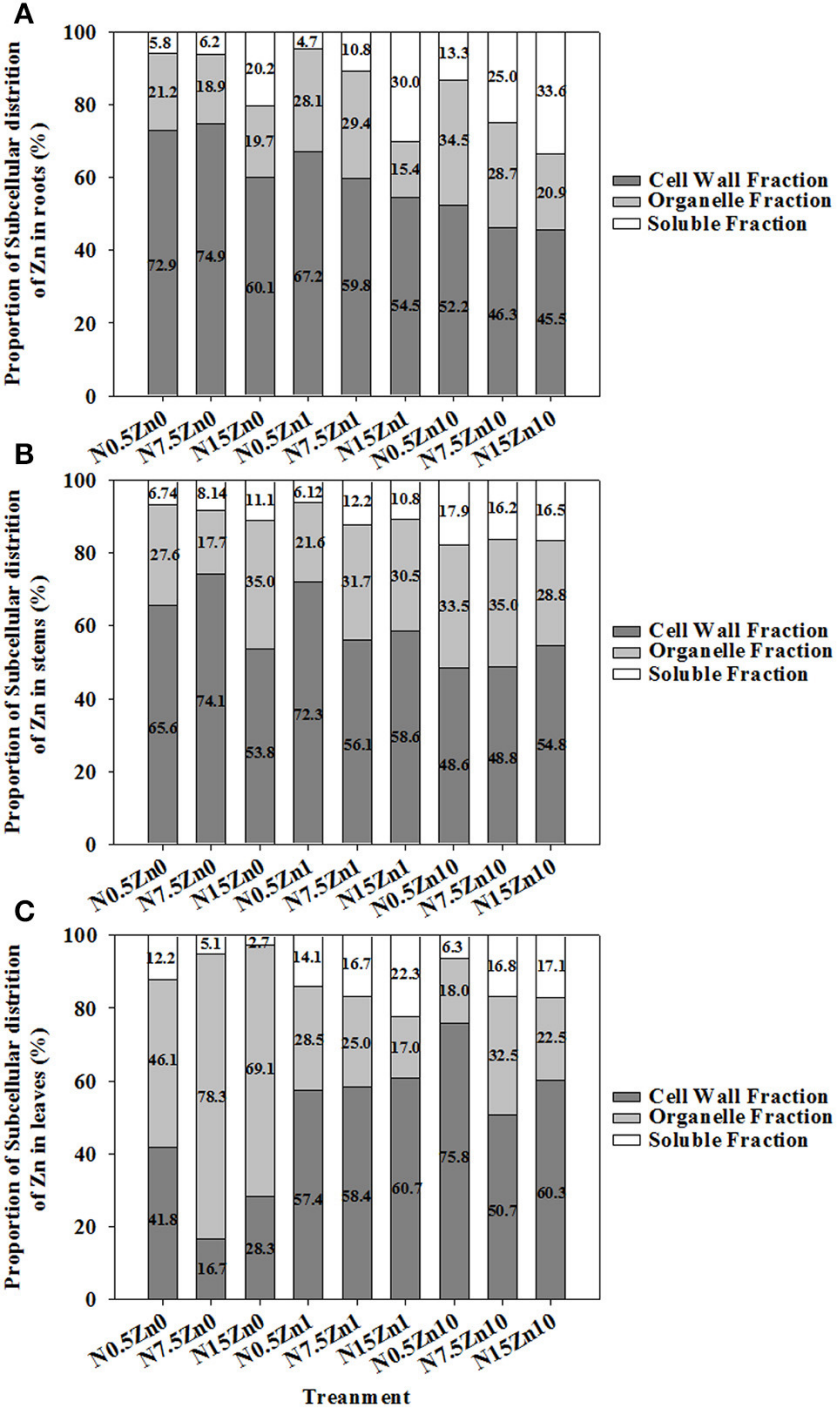
Subcellular distribution of Zn as a percent of total Zn in roots **(A)**, stems **(B)**, and leaves **(C)** of winter wheat (*Triticum aestivum* cv Yunong202) seedlings, grown at 0.5, 7.5, or 15 mmol N L^−1^ in a nutrient solution with 0, 1, and 10 μmol Zn L^−1^ supply for 21 d. Subcellular distribution of Zn (%) was calculated via dividing the fraction by the total of fractions in each tissue and multiplying the quotient by 100. Values are means of three independent replicates.

The proportion of Zn in the tissues of winter wheat was higher for cell walls than for cell organelles or the soluble fraction, except for a major portion of Zn that was found in leaf cell organelles in the Zn_0_ treatment (Figure 4). Two-way ANOVA revealed significant effects of N and Zn treatment, and N × Zn interaction on the proportion of Zn in some factions of tissues (*P* < 0.05 or *P* < 0.01; Table S5). For each plant part of winter wheat, a decreased proportion of Zn in cell organelles and an increased proportion of Zn in cell walls and soluble fractions were found with increasing Zn supply for each N treatment (Figure 4). The proportion of Zn in root cell organelles showed a strong decrease with an increase in N supply; however, an increased response was observed in root soluble fractions at both Zn_1_ and Zn_10_ (Figure 4A). At Zn_0_ and Zn_1_, an increase in N supply significantly increased the proportion of Zn in stem soluble fractions (Figure 4B). Zinc proportion in leaf cell walls in the N_0.5_ treatment was higher than that in the N_15_ or N_7.5_ treatments at Zn_0_ and Zn_10_ (Figure 4C). An inconsistent result was found for the proportion of Zn in leaf cell organelles.

## Discussion

### N_7.5_ combined with Zn_10_ increased Zn absorption through enhancing root growth in winter wheat

An increase in N supply positively contributed to root absorption and root-to-shoot transport of Zn; these findings concur with the results of Xue et al. (2012). However, the extent of influence of N supply on Zn concentration and accumulation in both shoots and roots was determined through differing levels of Zn application. Under Zn_0_, an increase in N supply decreased Zn concentrations in the shoots and roots of winter wheat (Figures 1A,B), which was attributed to a dilution effect caused by an increase in growth as a result of increased N availability (Alloway, 2008). Furthermore, N supply did not significantly contribute to Zn accumulation without an additional Zn supply (Figures 1C,D), which might be due to low concentrations of Zn in the solution, which in turn limited Zn absorption into the roots. For the Zn_1_ treatment, an increase in N supply increased Zn concentration and accumulation in shoots; however, it had no obvious effect in roots (Figure 1). This suggests that an increase in N supply positively impacted on the root-to-shoot transport of Zn and further Zn accumulation in shoots in Zn_1_. Enhancement of Zn concentration following an increase in N supply has been suggested due to the growth enhancing effect of N (Aciksoz et al., 2011). However, Xue et al. (2012) report that an increase in shoot growth caused by increased N supply cannot be the major reason for an increase in shoot Zn content. Our results suggested that the increased Zn concentration in shoots might be partially related to the enhanced growth that took place following an increase in N supply. On the one hand, Zn concentration and accumulation in roots were not affected by the N_15_ treatment (Figures 1B,D), though it significantly increased root dry weight (Table 1). On the other hand, dry weight and Zn concentrations of shoots were increased, and then Zn accumulation was enhanced by N supply (Table 1; Figures 1A,C). This might be because N supply enhanced Zn transfer from roots to shoots, even when a trace amount of Zn was in the solution. For Zn_10_, an increase in N supply increased Zn concentration and accumulation in both shoots and roots of winter wheat (Figure 1). These results showed that the positive effect of increasing N supply on Zn absorption and root-to-shoot transport became more and more remarkable as Zn application increased up to a high level. The results revealed that the combination of adequate or near-adequate N supply (N_7.5_ and N_15_) with high Zn supply (Zn_10_) was most beneficial to absorption and root-to-shoot transport of Zn than applications of N or Zn alone. Our previous study showed that N combined with a high Zn (30 kg Zn ha^−1^) treatment enhanced Zn absorption and translocation, and increased Zn accumulation in winter wheat grain that was grown in Zn-deficient soil (Zhao et al., 2016). Thus, in order to increase Zn concentration in wheat grain cultured on Zn-deficient soils, a combination of adequate N with high Zn application is a better choice.

The time-course depletion experiment also revealed the enhanced effect of increasing N supply on the Zn absorption kinetics in winter wheat. In the Zn_10_ treatment, the depletion of Zn from solution was significantly increased by N_7.5_ (Figure 3A). This suggests that the N_7.5_ treatment enhanced Zn removal from solution, and absorption through at root surface. The value of *V*_max_ was higher, and the values of *K*_m_ and *C*_min_ were lower for the N_7.5_ treatment, compared to the N_0.5_ and N_15_ treatments (Figures 3B–D). Therefore, enhanced Zn absorption in the N_7.5_ treatment might be related to the membrane transport systems regulating Zn absorption and transport processes. Erenoglu et al. (2011) also report that increasing N supply significantly enhances root Zn uptake, which is due to the N-enhanced abundance of transporters, including ZIPs such as iron-regulated transporter (IRT). Generally, the expression of transporters related to nutrient uptake is quick in response to the change of environmental factors (Nie et al., 2014), which explains why N supply over only 24 h was able to change Zn absorption in our study. Thus, investigating the effect of N supply on the transporter expression related to Zn absorption and translocation in winter wheat is deserving of further research.

In the current study, when N increased from N_7.5_ to N_15_, no further increase in Zn accumulation in shoots or roots was observed. Moreover, the N_7.5_ treatment significantly increased Zn concentrations in shoots and roots under Zn_1_ (shoots: N_7.5_ 34.11 mg kg^−1^ > N_15_ 23.40 mg kg^−1^; roots: N_7.5_ 48.26 mg kg^−1^ > N_15_ 33.71 mg kg^−1^) and Zn_10_ treatments (shoot: N_7.5_ 105.51 mg kg^−1^ > N_15_ 91.37 mg kg^−1^; root: N_7.5_ 310.61 mg kg^−1^ > N_15_ 236.07 mg kg^−1^; Figure 1). Similar results were obtained for differences in Zn depletion in solution and the values of *V*_max_, *K*_m_, and *C*_min_ between the N_7.5_ and N_15_ treatments (Figure 3). These results might be because the plants already had sufficient Zn under the N_7.5_ treatment.

Root morphology (root length, surface area, and volume) can reflect the growth conditions of plant roots as it is influenced by environmental factors (Fageria and Moreira, 2011). Our results showed that N_7.5_ could promote root development by increasing root length, surface area and volume at the Zn_10_ treatment. Xue et al. (2014) pointed that greater root lengths and surface area due to N application might contribute to the increase in Zn uptake of roots in winter wheat. Therefore, co-supply of N and Zn could stimulate roots development might be one reason why Zn absorption was enhanced. With a further increase in N supply to N_15_, root development of 7- and 14-d-old seedlings was inhibited, compared to the N_0.5_ treatment. However, compared to N_0.5_, N_15_ inhibited the growth of roots but still enhanced Zn absorption and accumulation in winter wheat, suggesting that interference with the Zn membrane transport process might be another reason for increasing Zn absorption following high rates of N application (Erenoglu et al., 2011).

In short, adequate N combined with high Zn supply increased *V*_max_ and decreased *K*_m_ and *C*_min_, and affected Zn membrane transport to enhance plant ability to absorb Zn. On the other hand, it increased root length, root surface area and root volume, and promoted root growth and development to enhance the absorption of Zn from soil solution.

### An increase in N supply was beneficial to a balanced distribution of Zn between subcellular fractions

In our study, the subcellular distribution of Zn was higher in cell walls than in cell organelles or soluble fractions in most treatments, except that a greater amount of Zn was present in the cell organelles of leaves in Zn_0_ (Table 3; Figure 4). It was speculated that winter wheat could transfer more Zn into cell organelles to maintain normal metabolism under Zn deficiency. Our results were consistent with results of Pan et al. (2016), who noted that, at toxic levels, the majority of Zn was localized in cell walls. This indicated that the cell wall was the primary site of Zn storage, whether Zn was present at sufficient or excessive levels in the soil.

An increase in N supply had diverse effects on the subcellular distribution of Zn in different tissues or at different levels of Zn application. In the Zn_0_ and Zn_1_ treatments, Zn distribution in root cell walls and cell organelles were significantly decreased by an increase in N supply (Table 3; Figure 4A). In contrast, an increase in N supply enhanced Zn distribution in the root soluble fraction. A decreased distribution of Zn in cell walls of roots and stems through an increase in N supply was beneficial to Zn translocation to shoots, as the accumulation of trace metals in cell walls would inhibit migration into the protoplast (Krzesłowska, 2011). An increase in N supply significantly increased Zn concentrations in each subcellular fraction in the Zn_10_ treatment (Table 3), which was related to the increased Zn concentrations seen with the high Zn treatment. However, an increase in N supply still decreased Zn distribution in root cell walls and cell organelles, while an increase occurred in root soluble fractions (Figure 4A). In leaves under the Zn_0_ treatment, an increase in N supply decreased the portion of Zn in cell walls and in the soluble fraction, and increased the portion of Zn in cell organelles (Figure 4C). This suggested that an increase in N supply could enhance Zn transport to cell organelles, thus maintaining a normal exchange of materials and signals between cells and the external environment when Zn is deficient (Hacisalihoglu and Kochian, 2003; Alloway, 2008). In Zn_1_ and Zn_10_, Zn concentrations, and distribution in soluble fractions were increased by an increase in N supply, indicating that N could enhance the compartmentalization of Zn in vacuoles, which are the main compartment for the soluble fraction (Weigel and Jäger, 1980).

## Conclusions

This study demonstrated that an adequate N supply enhanced Zn absorption and Zn accumulation in shoots. We found significant and positive correlations between N and Zn concentrations and accumulation. The results suggested that increased N supply positively contributed to root absorption and root-to-shoot transport of Zn. The N_7.5_ treatment significantly increased the value of *V*_max_, while it decreased the values of *K*_m_ and *C*_min_. A combination of N_7.5_ and Zn_10_ treatments stimulated root development by increasing root length, surface area, and volume of 14-d-old seedlings, and led to a change in root size from 7 to 14 d. This indicated that the positive effects of a combination of N_7.5_ and Zn_10_ on high affinity between the Zn uptake transporters and stimulated root development might contribute to Zn absorption and its accumulation in winter wheat. In roots, Zn distribution in cell walls and cell organelles was significantly decreased, and that in soluble fractions was increased by an increase in N supply. By contrast, in leaves, an increase in N supply significantly decreased Zn in cell walls and soluble fractions, and increased Zn in cell organelles under Zn_0_; however, it increased Zn distribution in the soluble fractions of Zn_1_ and Zn_10_. These results suggest that an increase in N supply enhanced Zn translocation to shoots, thus maintaining normal metabolism in winter wheat.

## Author contributions

HL conceived and designed the experiments. JW and JL performed the experiments. PZ analyzed the data. ZN wrote the paper.

### Conflict of interest statement

The authors declare that the research was conducted in the absence of any commercial or financial relationships that could be construed as a potential conflict of interest.

## Abbreviations

*V*_max_: maximum influx rate
*K*_m_: Michaelis constant
*C*_min_: minimum equilibrium concentrations
N: nitrogen
Zn: zinc.

## References

[B1] Aciksoz S. Yazici A. Ozturk L. Cakmak I. (2011). Biofortification of wheat with iron through soil and foliar application of nitrogen and iron fertilizers. Plant Soil 349, 215–225. 10.1007/s11104-011-0863-2

[B2] Alloway B. J. (2008). Zinc in Soils and Crop Nutrition, 2nd Edn. Brussels; Paris: IZA and IFA.

[B3] Barber S. A. (1979). Growth requirements for nutrients in relation to demand at the root surface, in The Soil-Root Interface, eds Harley J. L. Russell R. S. (London; New York, NY; San Francisco, CA: Academic Press), 5–20.

[B4] Bouis H. E. (2003). Micronutrient fortification of plants through plant breeding: can it improve nutrition in man at low cost? Proc. Nutr. Soc. 62, 403–411. 10.1079/PNS2003262 14506888

[B5] Cakmak I. (2008). Enrichment of cereal grains with zinc: agronomic or genetic biofortification? Plant Soil 302, 1–17. 10.1007/s11104-007-9466-3

[B6] Cakmak I. Pfeiffer W. H. McClafferty B. (2010). Review: biofortification of durum wheat with zinc and iron. Cereal Chem. 87, 10–20. 10.1094/CCHEM-87-1-0010

[B7] Chardonnens A. N. Wilma M. Vellinga S. Schat H. Verkleij J. A. Ernst W. H. (1999). Allocation patterns of zinc and cadmium in heavy metal tolerant and sensitive *Silene vulgaris*. J. Plant Physiol. 155, 778–787. 10.1016/S0176-1617(99)80096-0

[B8] Erenoglu E. B. Kutman U. B. Ceylan Y. Yildiz B. Cakmak I. (2011). Improved nitrogen nutrition enhances root uptake, root-to-shoot translocation and remobilization of zinc (65Zn) in wheat. New Phytol. 189, 438–448. 10.1111/j.1469-8137.2010.03488.x 21029104

[B9] Fageria N. Moreira A. (2011). The role of mineral nutrition on root growth of crop plants. Adv. Agron. 110, 251–331. 10.1016/B978-0-12-385531-2.00004-9

[B10] Guo J. Liu X. Zhang Y. Shen J. Han W. Zhang W.. (2010). Significant acidification in major Chinese croplands. Science 327, 1008–1010. 10.1126/science.1182570 20150447

[B11] Hacisalihoglu G. Kochian L. V. (2003). How do some plants tolerate low levels of soil zinc? Mechanisms of zinc efficiency in crop plants. New Phytol. 159, 341–350. 10.1046/j.1469-8137.2003.00826.x 33873363

[B12] Jiang T. H. Zheng S. J. Shi J. Q. Hu A. T. Shi R. H. (1995). Several considerations in kinetic research on nutrition uptake by plants. Plant Nutr. Ferti. Sci. 1, 11–17 (in Chinese with English abstract).

[B13] Krzesłowska M. (2011). The cell wall in plant cell response to trace metals: polysaccharide remodeling and its role in defense strategy. Acta Physiol. Plant. 33, 35–51. 10.1007/s11738-010-0581-z

[B14] Kutman U. B. Yildiz B. Ozturk L. Cakmak I. (2010). Biofortification of durum wheat with zinc through soil and foliar applications of nitrogen. Cereal Chem. 87, 1–9. 10.1094/CCHEM-87-1-0001

[B15] Le C. Zha Y. Li Y. Sun D. Lu H. Yin B. (2010). Eutrophication of lake waters in China: cost, causes, and control. Environ. Manage. 45, 662–668. 10.1007/s00267-010-9440-3 20177679

[B16] Li M. Wang S. X. Tian X. H. Zhao J. H. Li H. Y. Guo C. H. (2015). Zn distribution and bioavailability in whole grain and grain fractions of winter wheat as affected by applications of soil N and foliar Zn combined with N or P. J. Cereal Sci. 61, 26–32. 10.1016/j.jcs.2014.09.009

[B17] Li T. Q. Yang X. E. Yang J. Y. He Z. L. (2006). Zn accumulation and subcellular distribution in the Zn hyperaccumulator Sedum alfredii Hance. Pedosphere 16, 616–623. 10.1016/S1002-0160(06)60095-7

[B18] Liu Z. (1994). Regularities of content and distribution of zinc in soils of China. Sci. Agric. Sin. 27, 30–37 (in Chinese with English abstract).

[B19] Ma G. Jin Y. Li Y. Zhai F. Kok F. J. Jacobsen E.. (2008). Iron and zinc deficiencies in China: what is a feasible and cost-effective strategy? Public Health Nutr. 11, 632–638. 10.1017/S1368980007001085 17894916

[B20] Nie Z. J. Hu C. X. Liu H. E. Tan Q. L. Sun X. C. (2014). Differential expression of molybdenum transport and assimilation genes between two winter wheat cultivars (*Triticum aestivum*). Plant Physiol. Biochem. 82, 27–33. 10.1016/j.plaphy.2014.05.002 24880579

[B21] Pan X. Chen G. Shi C. Chai M. Liu J. Cheng S. (2016). Effects of Zn stress on growth, Zn accumulation, translocation, and subcellular distribution of Spartina alterniflora Loisel. Clean Soil Air Water 44, 579–585. 10.1002/clen.201400288

[B22] Peleg Z. Saranga Y. Yazici A. Fahima T. Ozturk L. Cakmak I. (2008). Grain zinc, iron and protein concentrations and zinc-efficiency in wild emmer wheat under contrasting irrigation regimes. Plant Soil 306, 57–67. 10.1007/s11104-007-9417-z

[B23] Pfeiffer W. H. McClafferty B. (2007). Harvest Plus: breeding crops for better nutrition. Crop Sci. 47(Suppl. 3), S88–S105. 10.2135/cropsci2007.09.0020IPBS

[B24] Rathore V. Bajaj Y. Wittwer S. (1972). Subcellular localization of zinc and calcium in bean (*Phaseolus vulgaris* L.) tissues. Plant Physiol. 49, 207–211. 10.1104/pp.49.2.207 16657926 PMC365930

[B25] Weigel H. J. Jäger H. J. (1980). Subcellular distribution and chemical form of cadmium in bean plants. Plant Physiol. 65, 480–482. 10.1104/pp.65.3.480 16661218 PMC440359

[B26] Welch R. M. Graham R. D. (2004). Breeding for micronutrients in staple food crops from a human nutrition perspective. J. Exp. Bot. 55, 353–364. 10.1093/jxb/erh064 14739261

[B27] Whatley F. Ordin L. Arnon D. I. (1951). Distribution of micronutrient metals in leaves and chloroplast fragments. Plant Physiol. 26:414. 10.1104/pp.26.2.414 16654381 PMC437510

[B28] White P. J. Broadley M. R. (2009). Biofortification of crops with seven mineral elements often lacking in human diets–iron, zinc, copper, calcium, magnesium, selenium and iodine. New Phytol. 182, 49–84. 10.1111/j.1469-8137.2008.02738.x 19192191

[B29] Xue Y. F. Yue S. C. Zhang Y. Q. Cui Z. L. Chen X. P. Yang F. C. (2012). Grain and shoot zinc accumulation in winter wheat affected by nitrogen management. Plant Soil 361, 153–163. 10.1007/s11104-012-1510-2

[B30] Xue Y. F. Zhang W. Liu D. Y. Yue S. C. Cui Z. L. Chen X. P. (2014). Effects of nitrogen management on root morphology and zinc translocation from root to shoot of winter wheat in the field. Field Crop. Res. 161, 38–45. 10.1016/j.fcr.2014.01.009

[B31] Yang J. Zhang J. (2006). Grain filling of cereals under soil drying. New Phytol. 169, 223–236. 10.1111/j.1469-8137.2005.01597.x 16411926

[B32] Zhao P. Yang F. Sui F. Wang Q. (2013). Effect of combined application of Zn and N fertilizers on nitrogen use, grain yield and protein content in winter wheat. J. China Agric. Univ. 18, 28–33. 10.3321/j.issn:1007-4333.2013.03.004. (Chinese with English abstract).

[B33] Zhao P. Yang F. Sui F. Wang Q. Liu H. (2016). Effect of nitrogen fertilizers on zinc absorption and translocation in winter wheat. J. Plant Nutr. 39, 1311–1318. 10.1080/01904167.2015.1106560

[B34] Zheng X. Han S. Huang Y. Wang Y. Wang M. (2004). Re-quantifying the emission factors based on field measurements and estimating the direct N2O emission from Chinese croplands. Global Biogeochem. Cycles 18, 60–70. 10.1029/2003GB002167

[B35] Zhuang Q. S. (2003). Chinese Wheat Improvement and Pedigree Analysis. Beijing: China Agriculture Press.

