# Supplementary material for: Absorption Kinetics and Subcellular Fractionation of Zinc in Winter Wheat in Response to Nitrogen Supply
Source: Front Plant Sci. 2017 Aug 18;8:1435. doi: 10.3389/fpls.2017.01435 (PMC5563362; doi:10.3389/fpls.2017.01435)
Supplement: Supplementary file 1 [file Table1.DOC]

**Supporting Information Tables S1–S5**

**Table S1** Two-way analysis of variance (ANOVA) of the effects of Zn, N treatment as well as their interactions on the dry weight, Zn and N concentrations and accumulation of winter wheat (*Triticum aestivum* cv Yunong202) grown under greenhouse conditions.

| Source of Variation (Treatment) |  | DF |  | Shoot Dry Weight | | |  | Root Dry Weight | | |  | Root / Shoot Ratio | | |
| --- | --- | --- | --- | --- | --- | --- | --- | --- | --- | --- | --- | --- | --- | --- |
|  |  |  |  | SS |  | F Pr. |  | SS |  | F Pr. |  | SS |  | F Pr. |
| Zn treatment |  | 2 |  | 0.064 |  | <.001 |  | 0.008 |  | <.001 |  | 0.016 |  | 0.014 |
| N treatment |  | 2 |  | 0.772 |  | <.001 |  | 0.019 |  | <.001 |  | 0.342 |  | <.001 |
| Zn x N |  | 4 |  | 0.026 |  | 0.036 |  | 0.003 |  | 0.035 |  | 0.015 |  | 0.088 |
| Source of Variation (Treatment) |  | DF |  | Shoot Zn Concentrations | | |  | Root Zn Concentrations | | |  | Shoot Zn Accumulations | | |
|  |  |  |  | SS |  | F Pr. |  | SS |  | F Pr. |  | SS |  | F Pr. |
| Zn treatment |  | 2 |  | 23561 |  | <.001 |  | 258892 |  | <.001 |  | 10550 |  | <.001 |
| N treatment |  | 2 |  | 3057.5 |  | <.001 |  | 10556.9 |  | <.001 |  | 3308.9 |  | <.001 |
| Zn x N |  | 4 |  | 5581.9 |  | <.001 |  | 22464.7 |  | <.001 |  | 3903.1 |  | <.001 |
| Source of Variation (Treatment) |  | DF |  | Root Zn Accumulations | | |  | Shoot N Concentrations | | |  | Root N Concentrations | | |
|  |  |  |  | SS |  | F Pr. |  | SS |  | F Pr. |  | SS |  | F Pr. |
| Zn treatment |  | 2 |  | 10120 |  | <.001 |  | 3.6737 |  | <.001 |  | 0.4035 |  | 0.006 |
| N treatment |  | 2 |  | 656.12 |  | <.001 |  | 13.218 |  | <.001 |  | 10.040 |  | <.001 |
| Zn x N |  | 4 |  | 1342.4 |  | <.001 |  | 0.4615 |  | 0.050 |  | 1.0733 |  | <.001 |
| Source of Variation (Treatment) |  | DF |  | Shoot N Accumulations | | |  | Root N Accumulations | | |  |  | | |
|  |  |  |  | SS |  | F Pr. |  | SS |  | F Pr. |  |  |  |  |
| Zn treatment |  | 2 |  | 298.30 |  | <.001 |  | 4.2025 |  | 0.005 |  |  |  |  |
| N treatment |  | 2 |  | 1300.7 |  | <.001 |  | 61.567 |  | <.001 |  |  |  |  |
| Zn x N |  | 4 |  | 93.167 |  | <.001 |  | 12.346 |  | <.001 |  |  |  |  |

**Table S2** One-way analysis of variance (ANOVA) of the effect of N treatment on the absorption kinetic parameters of winter wheat (*Triticum aestivum* cv Yunong202) grown under greenhouse conditions.

| Source of Variation (Treatment) |  | DF |  | V_max_ | | |  | K_m_ | | |  | C_min_ | | |
| --- | --- | --- | --- | --- | --- | --- | --- | --- | --- | --- | --- | --- | --- | --- |
|  |  |  |  | SS |  | F Pr. |  | SS |  | F Pr. |  | SS |  | F Pr. |
| N treatment |  | 2 |  | 137.64 |  | 0.016 |  | 519856 |  | 0.015 |  | 807922 |  | 0.013 |

**Table S3** Two-way analysis of variance (ANOVA) of the effects of Zn, N treatment as well as their interactions on the root morphological characteristics of winter wheat (*Triticum aestivum* cv Yunong202) grown under greenhouse conditions.

| Source of Variation (Treatment) |  | DF |  | Root Length  at 7d | | |  | Root Surface Area  at 7d | | |  |
| --- | --- | --- | --- | --- | --- | --- | --- | --- | --- | --- | --- |
|  |  |  |  | SS |  | F Pr. |  | SS |  | F Pr. |  |
| Zn treatment |  | 2 |  | 35898 |  | <.001 |  | 65.61 |  | 0.124 |  |
| N treatment |  | 2 |  | 53000 |  | <.001 |  | 2925 |  | 0.001 |  |
| Zn x N |  | 4 |  | 8849.9 |  | 0.238 |  | 319.8 |  | 0.004 |  |
| Source of Variation (Treatment) |  | DF |  | Root Volume  at 7d | | |  | Average Root Diameter  at 7d | | |  |
|  |  |  |  | SS |  | F Pr. |  | SS |  | F Pr. |  |
| Zn treatment |  | 2 |  | 0.0076 |  | 0.060 |  | 0.0005 |  | 0.101 |  |
| N treatment |  | 2 |  | 0.0118 |  | 0.017 |  | 0.0025 |  | <.001 |  |
| Zn x N |  | 4 |  | 0.0399 |  | <.001 |  | 0.0028 |  | 0.002 |  |
| Source of Variation (Treatment) |  | DF |  | Root Length  at 14d | | |  | Root Surface Area  at 14d | | |  |
|  |  |  |  | SS |  | F Pr. |  | SS |  | F Pr. |  |
| Zn treatment |  | 2 |  | 535577 |  | <.001 |  | 3090.5 |  | <.001 |  |
| N treatment |  | 2 |  | 981670 |  | <.001 |  | 3333.5 |  | <.001 |  |
| Zn x N |  | 4 |  | 220236 |  | 0.049 |  | 1592.5 |  | 0.008 |  |
| Source of Variation (Treatment) |  | DF |  | Root Volume  at 14d | | |  | Average Root Diameter  at 14d | | |  |
|  |  |  |  | SS |  | F Pr. |  | SS |  | F Pr. |  |
| Zn treatment |  | 2 |  | 0.0818 |  | <.001 |  | 0.0035 |  | 0.024 |  |
| N treatment |  | 2 |  | 0.0729 |  | <.001 |  | 0.0085 |  | <.001 |  |
| Zn x N |  | 4 |  | 0.1081 |  | <.001 |  | 0.0012 |  | 0.553 |  |

**Table S4** Two-way analysis of variance (ANOVA) of the effects of Zn, N treatment as well as their interactions on the Zn subcellular fractions of winter wheat (*Triticum aestivum* cv Yunong202) grown under greenhouse conditions.

| Source of Variation (Treatment) |  | DF |  | Root Cell Wall Fraction | | |  | Root Cell Organelle Fraction | | |  | Root Soluble Fraction | | |
| --- | --- | --- | --- | --- | --- | --- | --- | --- | --- | --- | --- | --- | --- | --- |
|  |  |  |  | SS |  | F Pr. |  | SS |  | F Pr. |  | SS |  | F Pr. |
| Zn treatment |  | 2 |  | 484.5 |  | <.001 |  | 289.6 |  | <.001 |  | 352.0 |  | <.001 |
| N treatment |  | 2 |  | 95.54 |  | 0.010 |  | 36.01 |  | <.001 |  | 103.9 |  | <.001 |
| Zn x N |  | 4 |  | 122.7 |  | 0.019 |  | 33.27 |  | <.001 |  | 93.19 |  | <.001 |
| Source of Variation (Treatment) |  | DF |  | Stem Cell Wall Fraction | | |  | Stem Cell Organelle Fraction | | |  | Stem Soluble Fraction | | |
|  |  |  |  | SS |  | F Pr. |  | SS |  | F Pr. |  | SS |  | F Pr. |
| Zn treatment |  | 2 |  | 129.0 |  | <.001 |  | 78.75 |  | <.001 |  | 35.46 |  | <.001 |
| N treatment |  | 2 |  | 120.1 |  | <.001 |  | 8.865 |  | 0.003 |  | 1.187 |  | 0.021 |
| Zn x N |  | 4 |  | 258.6 |  | <.001 |  | 12.35 |  | 0.004 |  | 0.972 |  | 0.142 |
| Source of Variation (Treatment) |  | DF |  | Leaf Cell Wall Fraction | | |  | Leaf Cell Organelle Fraction | | |  | Leaf Soluble Fraction | | |
|  |  |  |  | SS |  | F Pr. |  | SS |  | F Pr. |  | SS |  | F Pr. |
| Zn treatment |  | 2 |  | 277.9 |  | <.001 |  | 73.54 |  | <.001 |  | 15.69 |  | <.001 |
| N treatment |  | 2 |  | 4.056 |  | 0.014 |  | 50.25 |  | <.001 |  | 5.362 |  | 0.012 |
| Zn x N |  | 4 |  | 6.468 |  | 0.012 |  | 24.85 |  | 0.010 |  | 6.204 |  | 0.034 |

**Table S5** Two-way analysis of variance (ANOVA) of the effects of Zn, N treatment as well as their interactions on the relative distribution of Zn among subcellular fractions of winter wheat (*Triticum aestivum* cv Yunong202) grown under greenhouse conditions.

| Source of Variation (Treatment) |  | DF |  | Root Cell Wall Fraction | | |  | Root Cell Organelle Fraction | | |  | Root Soluble Fraction | | |
| --- | --- | --- | --- | --- | --- | --- | --- | --- | --- | --- | --- | --- | --- | --- |
|  |  |  |  | SS |  | F Pr. |  | SS |  | F Pr. |  | SS |  | F Pr. |
| Zn treatment |  | 2 |  | 1030 |  | <.001 |  | 146.5 |  | 0.046 |  | 408.1 |  | <.001 |
| N treatment |  | 2 |  | 265.6 |  | 0.042 |  | 209.7 |  | 0.016 |  | 944.0 |  | <.001 |
| Zn x N |  | 4 |  | 44.35 |  | 0.645 |  | 56.53 |  | 0.267 |  | 45.30 |  | 0.168 |
| Source of Variation (Treatment) |  | DF |  | Stem Cell Wall Fraction | | |  | Stem Cell Organelle Fraction | | |  | Stem Soluble Fraction | | |
|  |  |  |  | SS |  | F Pr. |  | SS |  | F Pr. |  | SS |  | F Pr. |
| Zn treatment |  | 2 |  | 496.8 |  | <.001 |  | 80.02 |  | 0.051 |  | 178.5 |  | <.001 |
| N treatment |  | 2 |  | 94.38 |  | 0.077 |  | 39.15 |  | 0.207 |  | 15.95 |  | 0.028 |
| Zn x N |  | 4 |  | 240.8 |  | <.001 |  | 155.3 |  | 0.002 |  | 16.17 |  | 0.012 |
| Source of Variation (Treatment) |  | DF |  | Leaf Cell Wall Fraction | | |  | Leaf Cell Organelle Fraction | | |  | Leaf Soluble Fraction | | |
|  |  |  |  | SS |  | F Pr. |  | SS |  | F Pr. |  | SS |  | F Pr. |
| Zn treatment |  | 2 |  | 3031 |  | <.001 |  | 4936 |  | <.001 |  | 279.3 |  | <.001 |
| N treatment |  | 2 |  | 607.5 |  | <.001 |  | 477.6 |  | <.001 |  | 23.42 |  | 0.209 |
| Zn x N |  | 4 |  | 178.0 |  | 0.003 |  | 308.9 |  | 0.010 |  | 108.9 |  | <.001 |
